# Regulation of IgE activity in inhalational tolerance via formation of IgG anti-IgE/IgE immune complexes

**DOI:** 10.1186/s12948-018-0091-x

**Published:** 2018-05-18

**Authors:** Sonali J. Bracken, Alexander J. Adami, Ektor Rafti, Craig M. Schramm, Adam P. Matson

**Affiliations:** 1 Department of Immunology, University of Connecticut Health, 263 Farmington Avenue, Farmington, CT 06030 USA; 2 Department of Pediatrics, Connecticut Children’s Medical Center, Hartford, CT USA

**Keywords:** Anti-IgE, Asthma, Autoantibodies, Hexosaminidase, IgE, Omalizumab, Ovalbumin, Tolerance

## Abstract

**Background:**

Allergic asthma is an inflammatory disorder of the airways that results from inappropriate production of IgE against harmless, environmental antigens. Sequestration of free IgE using humanized IgG anti-IgE is an effective therapy for asthma and other atopic disorders. However, the status of free IgE in subjects who have naturally developed immune tolerance to inhaled antigens has not been well studied.

**Methods:**

C57BL/6 mice were sensitized and challenged with ovalbumin (OVA) for 7 days to induce allergic airway disease (AAD) or 6 weeks to induce a state of local inhalational tolerance (LIT). Serum from AAD or LIT mice, diluted to achieve equivalent levels of total OVA-specific IgE, was used to sensitize rat basophil leukemia cells for allergen-mediated degranulation. Levels of degranulation were measured in relation to serum concentrations of free IgE and IgG anti-IgE/IgE immune complexes.

**Results:**

Serum from AAD animals induced a greater degree of basophil degranulation than serum from LIT animals. These results correlated with higher levels of free IgE in AAD animals, whereas LIT mice demonstrated a significant increase in IgG anti-IgE/IgE immune complexes relative to their diseased counterparts.

**Conclusions:**

Sequestration of free IgE by naturally occurring IgG anti-IgE may aid in the development of immune tolerance against inhaled allergens. The decrease in bioavailability of free IgE may, in turn, contribute to the overall reduction of asthma symptoms via a mechanism that mimics the therapeutic effects of humanized IgG anti-IgE.

## Background

Allergic asthma is an IgE-mediated hypersensitivity response that results from inappropriate immune activation (i.e. lack of immune tolerance) against innocuous environmental allergens [1]. Current treatments for asthma are primarily geared towards symptom control and have not been successful in reducing overall prevalence of this condition. The sole exception is allergen-specific immunotherapy (ASIT), a treatment modality where repeated exposure to the triggering allergen is used to induce tolerance to that allergen. Due to its disease-modifying effects, ASIT has the potential to alter the progression of allergic asthma, yet the multiple mechanisms involved in this treatment modality and optimal route of allergen administration remain to be fully elucidated [2]. Understanding the factors that contribute to development of immune tolerance against inhaled allergens is imperative for improving our current asthma treatment strategies.

Previous studies from our laboratory have shown that mice develop allergic airway disease (AAD) after short-term challenge with the model antigen ovalbumin (OVA), whereas long-term challenge results in the development of local inhalational tolerance (LIT) against OVA with subsequent resolution of the disease [3]. Interestingly, we have noted that levels of OVA-specific IgE are considerably higher in LIT mice when compared to AAD mice despite resolution of local eosinophilia, inflammation, and airway hyper-responsiveness in the former group [4]. A similar discrepancy between IgE levels and severity of allergic disease is observed in individuals receiving omalizumab, a recombinant humanized IgG anti-IgE intended for patients with moderate-to-severe asthma. In such cases, omalizumab rapidly sequesters free IgE, thereby reducing the potential for degranulation of FcεRI^+^ cells. However, total IgE levels remain elevated or may even increase following initiation of therapy due to the slow clearance of biologically inactive omalizumab-IgE complexes [5]. This led us to question whether altered levels of free IgE may play a role in the phenotypic differences between mice with active OVA-induced AAD and those that have become tolerant to OVA.

## Methods

Female C57BL/6 mice (6–8 per group), 6–8 weeks of age, were purchased from Jackson Laboratory (Bar Harbur, ME) and were housed and cared for as previously described [3]. All animal protocols were approved by the Animal Care Committee at the University of Connecticut Health. Mice were sensitized with 3 weekly intraperitoneal injections of a suspension containing 25 µg OVA (Sigma-Aldrich, St. Louis, MO) and 2 mg of aluminum hydroxide [3]. After the last injection, mice were exposed to a 1% OVA aerosol solution for 1 h daily for a total of either 7 days (AAD) to induce a state of disease or 6 weeks (LIT) to induce a state of tolerance. Mice were sacrificed 24 h after the last OVA exposure and serum was obtained via cardiac puncture.

The biologic activity of IgE in serum samples was determined using a rat basophil mediator release assay [6]. RBL-2H3 cells (American Type Culture Collection, Manassas, VA) were preincubated for 1 h at 37 °C with four serial dilutions of AAD or LIT serum, at equivalent concentrations of OVA-specific IgE as determined by ELISA [4]. Naïve samples containing undetectable levels of OVA-specific IgE were added as two-fold serial dilutions (range: 1/1 to 1/8) as negative control serum. Degranulation was induced by adding OVA (5 µg/ml) in Tyrode’s salt solution for 1 h at 37 °C. Total cell release was obtained by adding 1% Triton x-100 to positive control samples. Results were expressed as a percentage of total release.

Levels of IgG_1_ anti-IgE/IgE immune complexes were determined as previously described [6]. For determination of free IgE levels, 96-well plates were coated with 2 μg/ml recombinant human high affinity IgE receptor, α-chain (NBS-C Bioscience, Vienna, Austria), which binds to both human and murine IgE [7]. Serum samples were added based on equivalent concentrations of total IgE as determined by ELISA (2). Standard curves were generated using mouse IgE kappa anti-TNP (C38-2; BD Pharmingen, Franklin Lakes, NJ). After washing, HRP-conjugated goat-anti mouse IgE (A1-H12; Southern Biotech, Birmingham, AL) was applied at a 1:8000 concentration. Plates were developed with the TMB microwell peroxidase substrate system and measured with a microplate reader at a dual absorbance of 450 and 570 nm.

All statistical analyses between groups were made via one-way analysis of variance (ANOVA) followed by Newman–Keuls post hoc test. β-hexosaminidase release data were compared via area under the curve analysis. Data were expressed as mean ± SEM values for all comparisons; values of *P* ≤ 0.05 were used as the threshold for significance. All statistical analysis was performed using the GraphPad Prism (La Jolla, Calif., USA) statistical software package.

## Results

**Fig. 1:**
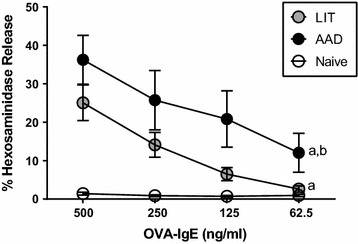
LIT serum demonstrates a reduced capacity to elicit RBL mediator release compared to AAD serum. RBL assays were performed as described above. For AAD and LIT samples, twofold serial dilutions starting at 500 ng/ml OVA-IgE were performed. For naïve animals, samples were diluted using twofold serial dilutions from 1:1 to 1:8 concentrations. β-hexosaminidase release was measured as a percentage of total release in control samples. n = 6–8 per group. ^a^p < 0.05 vs naïve, ^b^p < 0.05 vs LIT

As previously demonstrated by our laboratory [4], serum levels of OVA-specific IgE were significantly higher in LIT mice (31,609 ± 7351 ng/ml) relative to AAD mice (5647 ± 2004 ng/ml), *P* < 0.001. OVA-specific IgE levels were virtually undetectable in naïve controls (0.1 ± 0.1 ng/ml). When evaluating the biological activity of the OVA-specific IgE using RBL cells, both AAD and LIT serum led to a greater degree of β-hexosaminidase release than naïve serum; however, the mediator release generated using AAD serum was significantly greater than that observed using LIT serum (Fig. 1). These results indicate that despite equivalent amounts of OVA-specific IgE being added to RBL cells, the AAD serum led to a greater degree of sensitization and degranulation than LIT serum.

**Fig. 2:**
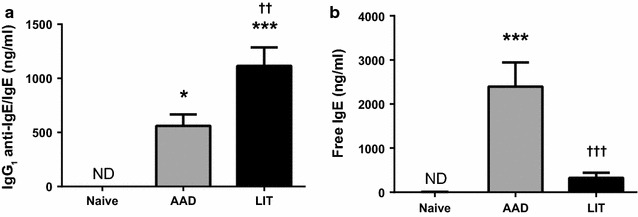
LIT serum contains higher levels of IgG anti-IgE/IgE immune complexes and lower free IgE levels relative to AAD serum. **a** Levels of IgG_1_ anti-IgE/IgE immune complexes were determined as described above. For naïve samples, undiluted serum was used. **b** Levels of free IgE were determined in samples with equal concentrations of total IgE as described above. For naïve samples, undiluted serum was used. n = 6–8 per group. *p < 0.05 vs naïve, ***p < 0.001 vs naïve, ^††^p < 0.01 vs AAD, ^†††^p < 0.001 vs AAD. ^ND^ none detected

To evaluate a potential cause for the reduced bioactivity of LIT serum, levels of IgG_1_ anti-IgE/IgE immune complexes were measured in serum obtained from all three groups. No immune complexes were detected in naïve animals (Fig. 2a). In contrast, IgG_1_ anti-IgE/IgE immune complexes were present in the serum of AAD mice but were significantly higher in LIT mice (*P* < 0.01). This suggested that there was more non-complexed IgE (free IgE) in the serum of AAD mice. To confirm this possibility, free IgE levels were measured in the serum samples using recombinant FcεRIα protein in a method similar to that used to measure free IgE in humans [5]. Naïve animals had undetectable levels of free IgE, whereas AAD mice had a significantly higher concentration of free IgE when compared to naïve controls (*P* < 0.001). However, concentrations of free IgE did not statistically differ between LIT mice and controls (Fig. 2b). Moreover, there was a significant decrease in free IgE levels from AAD to LIT (*P* < 0.001), which may account for the decrease in RBL mediator release observed in the latter group.

## Discussion

Naturally occurring autoantibodies directed against IgE are known to exist in a large proportion of subjects with atopic disease [8]; however, their precise role in the regulation of allergic responses remains unclear. The development and increasing use of omalizumab has provided some insight into how naturally occurring IgG anti-IgE antibodies with similar epitope specificity may serve to limit IgE activity. In subjects treated with omalizumab, the reduction in free IgE levels corresponds with improvement in allergic symptomatology, yet total IgE levels [5] and allergen-specific IgE levels [9] remain elevated or even increased, as standard assays do not differentiate between free IgE and IgE bound to IgG anti-IgE. We observed a similar discrepancy between IgE levels and the resolution of allergic disease in mice subjected to LIT and therefore investigated whether free IgE levels would be altered with tolerance development. Our findings suggest that immune tolerance to inhaled allergens is associated with increased levels of IgG_1_ anti-IgE/IgE immune complexes and a reduction in free IgE. Furthermore, these changes are linked to a diminished capacity of IgE to sensitize FcεRI-expressing cells such as basophils and mast cells. Thus, the importance of IgE may lie less in its absolute concentration and more in its capacity to bind FcεRI (free IgE).

The formation of IgG_1_ anti-IgE/IgE immune complexes in LIT is particularly noteworthy as the efficacy of allergen-specific immunotherapy has been shown to rely partially upon the induction of IgG antibody subclasses that are capable of modulating IgE responses through direct blocking effects as well as through binding to inhibitory FcγRIIb receptors [2]. In particular, human IgG4, a similar subtype to mouse IgG1, has been shown to increase during ASIT administration [10], and at least one group has shown increases in human IgG4 during development of spontaneous tolerance to cow’s milk [11]. That IgG4 could be the controlling factor for tolerance despite persistence of high IgE could open up new avenues for therapy to augment ASIT and traditional asthma treatment. The possibility that high serum levels of OVA-specific IgG in LIT mice [4] contributed to the reduction in RBL mediator release should also be considered; however, the presence of such antibodies would not alter the assay findings of increased complex formation and reduced free IgE. Additional studies are required to determine the contribution of OVA-specific IgG in this model system and whether IgG anti-IgE/IgE immune complexes serve additional tolerogenic roles in the context of asthma or allergen-specific immunotherapy, which may include effects on regulatory cells [12] and co-engagement of FcεRI with FcγRIIb [13].

## Conclusions

Results from this study suggest that immune tolerance to inhaled allergens may be partially mediated by formation of IgG_1_ anti-IgE/IgE immune complexes. Such changes may lead to a decreased level of free IgE, which reduces the potential for IgE to sensitize FcεRI-expressing cells such as basophils and mast cells. Presumably, such changes in the bioactivity of IgE would reduce overall symptomatology of allergic asthma in a manner that is analogous to the therapeutic administration of humanized IgG anti-IgE.

## Abbreviations

OVA: ovalbumin
AAD: allergic airway disease
LIT: local inhalational tolerance
RBL: rat basophil leukemia
